# MET amplification in metastatic colorectal cancer: an acquired response to EGFR inhibition, not a *de novo* phenomenon

**DOI:** 10.18632/oncotarget.10559

**Published:** 2016-07-13

**Authors:** Kanwal Raghav, Van Morris, Chad Tang, Pia Morelli, Hesham M. Amin, Ken Chen, Ganiraju C. Manyam, Bradley Broom, Michael J. Overman, Kenna Shaw, Funda Meric-Bernstam, Dipen Maru, David Menter, Lee M. Ellis, Cathy Eng, David Hong, Scott Kopetz

**Affiliations:** ^1^ Department of Gastrointestinal Medical Oncology, The University of Texas MD Anderson Cancer Center, Houston, TX, USA; ^2^ Department of Radiation Oncology, The University of Texas MD Anderson Cancer Center, Houston, TX, USA; ^3^ Department of Hematopathology, The University of Texas MD Anderson Cancer Center, Houston, TX, USA; ^4^ Department of Bioinformatics and Computational Biology, The University of Texas MD Anderson Cancer Center, Houston, TX, USA; ^5^ Sheikh Khalifa Bin Zayed Al Nahyan Institute of Personalized Cancer Therapy, The University of Texas MD Anderson Cancer Center, Houston, TX, USA; ^6^ Department of Cancer Therapeutics, The University of Texas MD Anderson Cancer Center, Houston, TX, USA; ^7^ Department of Surgical Oncology, The University of Texas MD Anderson Cancer Center, Houston, TX, USA

**Keywords:** amplification, MET, circulating-free DNA, fluorescence in situ hybridization, colorectal cancer

## Abstract

**Background:**

MET amplification appears to be a predictive biomarker for MET inhibition. Prior studies reported a MET amplification rate of 9–18% in metastatic colorectal cancer (mCRC) but do not differentiate increased gene copy numbers due to chromosomal level aberrations from focal gene amplifications. Validation of MET amplification rate in mCRC is critical to this field.

**Results:**

In tumor tissue-based analyses, overall MET amplification rate was 1.7% (10/590). MET amplification was seen in 0/103 (0%), 4/208 (1.9%) and 6/279 (2.2%) cases, in cohorts 1, 2 and 3, respectively. Rate of MET amplification in cfDNA of cohort 4 patients refractory to anti-EGFR therapy (*n* = 53) was 22.6% (12/53) and was significantly higher compared to patients not exposed to anti-EGFR therapy (*p* < 0.001).

**Materials and Methods:**

We analyzed MET amplification in mCRC (*n* = 795) using different methods across multiple cohorts. Cohort 1 (*n* = 103) and 2 (*n* = 208) included resected liver metastases and tumor biopsies, respectively, tested for MET amplification using fluorescence *in-situ* hybridization [amplification: MET/CEP7 ratio ≥ 2.0]. Using another tissue-based approach, cohort 3 (*n* = 279) included tumor biopsies sequenced with HiSeq (Illumina) with full exome coverage for MET [amplification: ≥ 4 copies identified by an in-house algorithm]. Using a blood-based approach by contrast, cohort 4 (*n* = 205) included patients in whom the full exome of MET in circulating-free DNA (cfDNA) was sequenced with HiSeq.

**Conclusions:**

Contrary to prior reports, in this large cohort, MET amplification was a rare event in mCRC tissues. In plasma by stark contrast, MET amplification identified by cfDNA occurred in a sizable subset of patients that are refractory to anti-EGFR therapy.

## INTRODUCTION

MET (mesenchymal-epithelial transition factor) proto-oncogene on chromosome 7q31 encodes for a receptor tyrosine kinase (RTK) and regulates a variety of downstream signaling pathways that initiate gene expression involved in promoting tumor growth, survival, angiogenesis, invasion and metastases. [1, 2] Due to its critical role in cancer biology, inhibition of the MET pathway is being actively investigated in numerous clinical trials. [1–4] MET amplification appears to identify a subset of cancers, uniquely sensitive to MET inhibitors, both *in vitro* and *in vivo*. [2, 5–7] MET amplification also drives resistance to anti-epidermal growth factor receptor (EGFR) monoclonal antibodies in colorectal cancer. [8] Consequently, MET amplification could potentially serve as a useful predictive biomarker of MET inhibitor response in mCRC clinical trials. Unfortunately, limited data exists regarding the prevalence of MET amplification in mCRC. [9] The most cited report assessed MET amplification using a quantitative polymerase chain reaction (PCR)-based assay (*N* = 217) and described an amplification rate of 9% in primary lesions and 18% in liver metastases. [9] However, these PCR-based assays were unable to differentiate between increased copy numbers from chromosomal level aberrations from focal gene amplification as is evident from studies in gastric cancer. [5, 6].

In this study, we examined a large number samples from mCRC cases across multiple cohorts to identify the frequency of MET amplification as determined by different methodologies along with a novel exploratory determination of MET amplifications in circulating cell-free DNA.

## RESULTS

### MET amplification in tumor tissue-based biopsies

**Figure 1:**
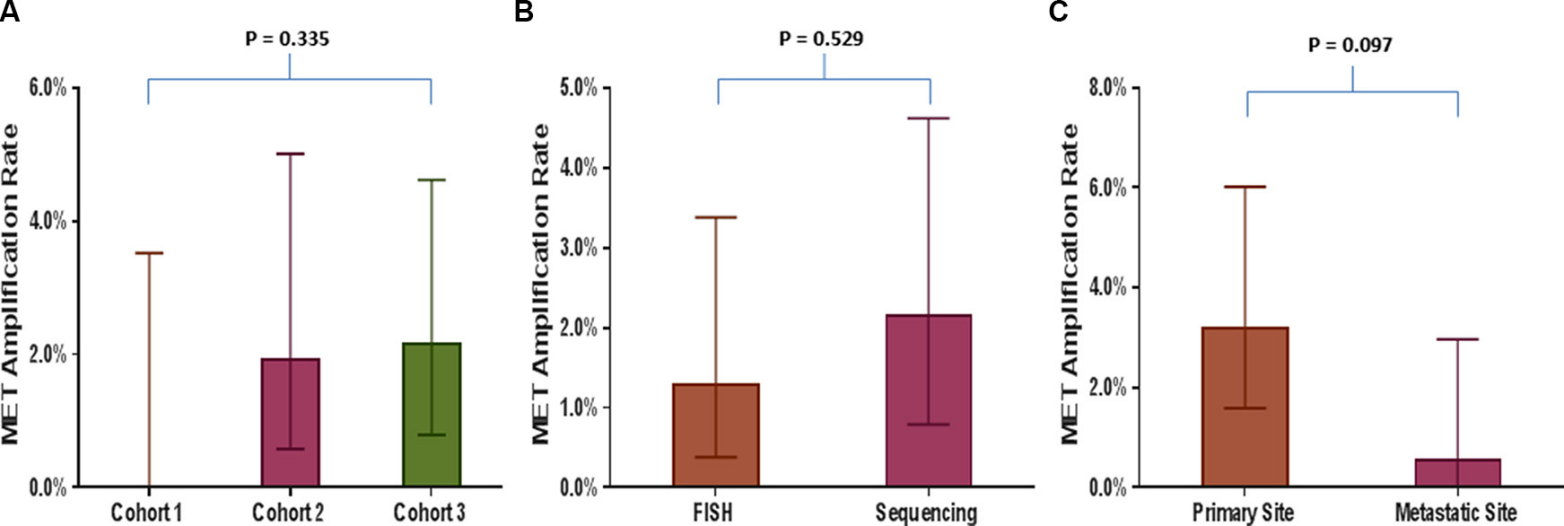
Comparison of MET amplification rate in various tumor tissue based analyses Bar graphs comparing MET amplification rate between (**A**) Different cohorts of patients with tumor tissue-based analyses (cohort 1 vs. 2 vs. 3); (**B**) Two methodologies used to assess MET amplification, fluorescence *in situ* hybridization (FISH) and sequencing; (**C**) Primary and metastatic site.

MET amplification was seen in 10 (1.7%; 95% CI: 0.01–3.14%) of 590 tumor tissue biopsies tested by both FISH and sequencing. MET amplification using FISH was seen in 0/103 (0.0%; 95% CI: 0.00–4.32%) and 4/208 (1.9%; 95% CI: 0.58–5.01%) cases in cohorts 1 and 2, respectively (MET/CEP7 ratio: 2.0–7.7). MET amplification using sequencing was seen in 6/279 (2.2%; 95% CI: 0.01–4.72%) (MET gene copy numbers (GCN): 4.0–6.7) (Table 1). There was no significant difference among proportion of MET amplification between different cohorts (*p* = 0.34), FISH and sequencing (*p* = 0.53) and primary (3.2%; 95% CI: 1.6–6.0%) and metastatic sites (0.5%; 95% CI: 0.0–3.3%) (*p* = 0.097) (Figure 1A–1C). Mutations in TP53 gene were the most common concurrent mutations seen in these patients (Supplementary Table S1).

**Table 1:** MET amplification proportion in multiple cohorts of mCRC

Study of Tissue Based Biopsies							
Cohort	N	Primary Site	Metastatic Site	Relationship of Samples to Prior Systemic Therapy	Test Employed for MET Amplification	Proportion of MET Amplified Cases	Rate of MET Amplification 95% CI
1^a^	103	NA	103	Pre-treatment	FISH	0 (0.0%)	0.0%–4.3%
2^a^	208	130	75	Pre-treatment	FISH	4 (1.9%)	0.6%–5.0%
3^a^	279	161	110	Pre-treatment	Sequencing	6 (2.2%)	0.9%–4.7%

Study of Blood Based Biopsies							
Cohort	N	Sub Cohort N	RAS Status	Prior Treatment & Progression on Anti-EGFR Therapy	Test Employed for MET Amplification	Proportion of MET Amplified Cases	Rate of MET Amplification 95% CI
4	205	4A (53)	RAS-WT	Yes	Sequencing	12 (22.6%)	13.3%–35.7%
		4B (43)	RAS-WT	No		2 (4.7%)	0.4%–16.3%
		4C (109)	RAS-Mut	NA		4 (3.7%)	1.1%–9.4%

**Abbreviations:** CI, confidence interval; EGFR, epidermal growth factor receptor; FISH, fluorescence *in-situ* hybridization; N, number of patients; NA, not applicable; Mut, mutated; PCR, polymerase chain reaction; WT, wild-type.

a Cohort 1 has only liver metastases; Site of the biopsy was unknown in 3 and 8 cases in Cohorts 2 and 3, respectively.

### MET amplification in blood-based biopsies (cfDNA)

**Figure 2:**
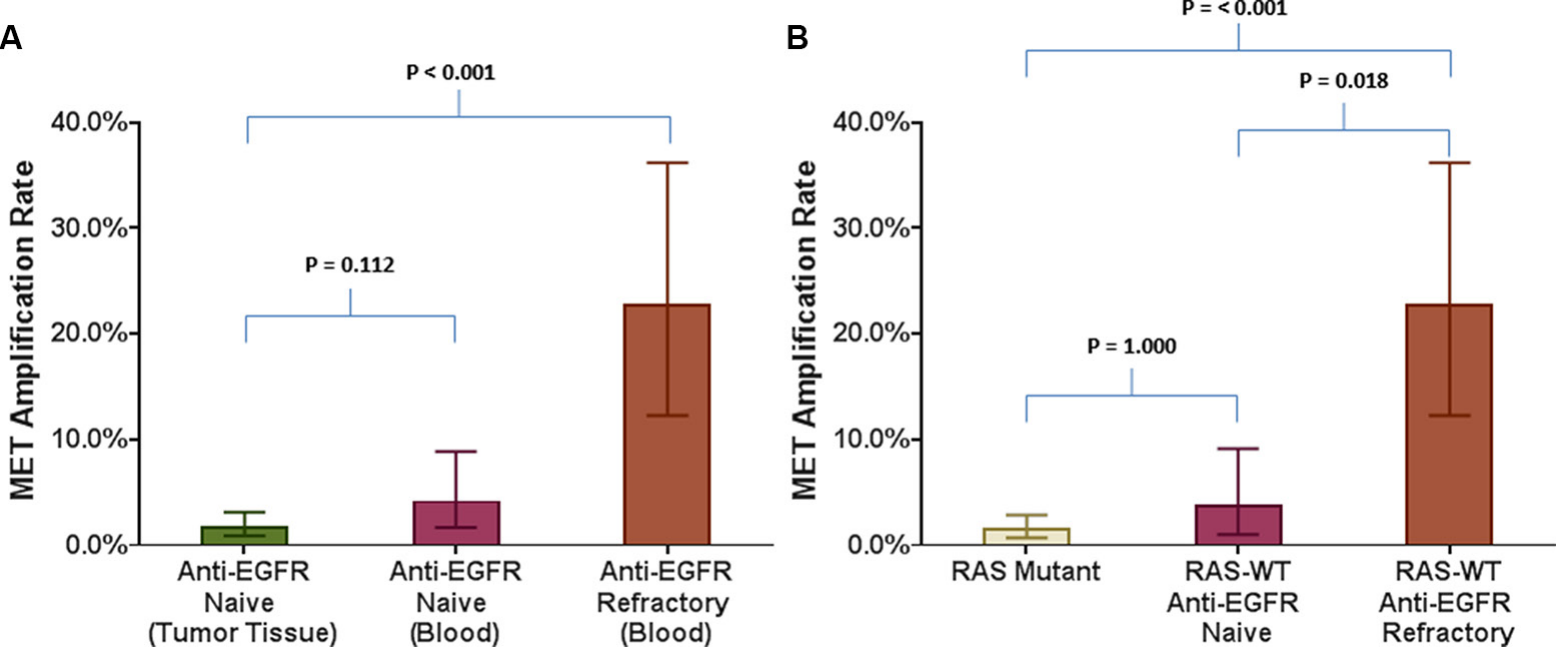
Comparison of MET amplification rate in various tumor tissue based and blood based analyses in relation to refractoriness to anti-EGFR therapy Bar graphs comparing MET amplification rate between (**A**) Anti-EGFR naïve tumor tissue biopsies and blood of anti-EGFR refractory RAS wild type patients; (**B**) Blood from RAS mutant patients and RAS wild type patients who are either anti-EGFR naïve or refractory to anti-EGFR therapy.

In cohort 4, 53 RAS wild-type patients had been previously treated with and had disease progression on anti-EGFR therapy prior to collection of plasma. MET amplification in this anti-EGFR therapy refractory cohort was detected on cfDNA in 12 (22.6%; 95% CI: 13.31–35.67%) cases (Table 1). This proportion was significantly higher compared to MET amplification seen in anti-EGFR naïve tumor tissue-based biopsies (*p* < 0.001) (Figure 2A). Furthermore, this rate was also significantly higher compared to the rate of MET amplification seen in cfDNA of either RAS mutated patients (*p* < 0.001) or RAS wild-type tumors without prior anti-EGFR antibody exposure (*p* = 0.018) (Figure 2B). No difference in rate of cfDNA MET amplification was evident with other intervening therapies (Supplementary Figure S1).

## DISCUSSION

In this large cohort of mCRC patients, we failed to validate the high prevalence of MET amplification in tissue samples as reported in prior studies with either FISH or sequencing. [9] Contrary to these reports, we observed that MET amplification is rare (1–2%) in mCRC (as opposed to 9–18%) and is not different between primary and metastatic lesions. [9] Our findings are consistent with the somatic copy-number alteration data generated by The Cancer Genome Atlas (TCGA) wherein only 1 case of high-level MET amplification was seen in a total of 276 colorectal tumors. [10, 11] We therefore believe that this study more accurately represents the incidence of MET amplification in *de novo* mCRC.

Discrepancies between our study and others can be explained by the limitations of PCR-based assays in detecting copy number. Both gene amplification and polysomy can result in increased GCNs in tumors. Experience in breast cancer reveals these two phenomena to be mechanistically distinct. Cases with Chromosome-17 (site of HER2/neu gene) polysomy compared to focal gene amplification of HER2, have lower GCNs, lower level of HER2 protein expression, features more consistent with HER2 negative tumors and do not derive any benefit from HER2-targeted treatment. [12, 13] Similar discrepancy in rate of MET gene amplification was also observed in both gastric and lung cancer. [5, 6].

Gain of 7q without any focal amplifications in MET in the TCGA cohort further supports for our findings. [11] Likewise, in a study using real-time quantitative PCR with transcript normalization no focal high-grade amplification of MET was seen in 103 liver metastases in mCRC but low level chromosome 7 polysomy was seen in 22 (21%) cases. [14] MET inhibition showed no effect on tumor growth on patient-derived xenografts chromosome 7 polysomy cases, irrespective of MET expression. [14] We therefore conclude that MET amplification is a very low prevalence tissue biomarker in *de novo* mCRC and therefore a difficult target for enrichment trials with MET targeted therapy. We also recommend FISH or NGS with adequate coverage as ideal tests for assessment of MET amplification in mCRC.

Despite the low prevalence of MET amplification in mCRC, we found that a substantial subset (23%) of patients with mCRC, who have been treated and are refractory to anti-EGFR antibodies, acquire MET amplification, as detected in cfDNA. Bardelli et al. showed that acquired MET amplification in tumor tissue is associated with acquired resistance to anti-EGFR antibodies in patients of mCRC. [8] Our findings indicate similar phenomenon and suggest the possibility of detecting MET amplification mediated resistance to anti-EGFR therapies using cfDNA. In patients with lung cancer, high level of consistency is seen between peripheral blood and tissue with regards to MET gene amplification. [15] We therefore propose that cfDNA could be a potential blood-based option in lieu of repeated post-treatment tissue biopsies for assessing acquisition of MET amplified phenotype in mCRC. However, it should be noted that additional mutations that could be associated with acquired resistance to anti-EGFR therapy were also seen with MET amplifications in cfDNA (Supplementary Tables S1 and S2). It is uncertain if MET amplification is the sole driver of resistance in these cases, and this heterogeneity may be a barrier to therapeutic interventions to target MET after acquisition of resistance to EGFR inhibition.

Future efforts should focus on refining evaluation of MET amplification. Using FISH for amplification is not without its drawbacks. Since FISH assessed increase in CEP7 copies > 3 may reflect either true polysomy or centromeric amplification, newer methodologies, such as multiplex ligation-dependent probe amplification that allow simultaneous quantification of multiple loci, can help detect focal MET amplifications that are missed by FISH and may be effective adjunct evaluation in clinical trials. [16] Furthermore, other unique molecular aberration, such as MET exon 14 deletion which can lead to MET overexpression, also need further investigations. [17] Efforts using serial cfDNA analyses are needed to determine whether these acquired MET amplifications are transitory or perpetual. Additionally, to fully comprehend the nature of acquired resistance to anti-EGFR therapy, the role of molecular alterations that may be acquired concurrently with MET amplifications needs to be investigated.

## MATERIALS AND METHODS

### Patients

We performed systematic analyses of 795 mCRC patients, at MD Anderson Cancer Center, Houston, Texas, U.S.A, between January 2010 and September 2015, across 4 different cohorts, who underwent MET amplification testing by various methods. Cohort 1 (*n* = 103) comprised of newly diagnosed mCRC patients with resected liver metastases. Cohorts 2 (*n* = 208) and 3 (*n* = 279) contained refractory mCRC patients with pre-treatment tumor tissue biopsies (both primary and metastatic sites). [3] Cohort 4 (*n* = 205) included mCRC patients with prospectively collected plasma after at least one-prior line of therapy (Table 1) (see details in eMethods in Supplement).

### Samples and MET amplification testing

In cohort 1 and cohort 2, pre-treatment formalin-fixed paraffin embedded (FFPE) tumor tissue was tested using fluorescence *in-situ* hybridization (FISH). Focal MET amplification was defined as MET/CEP7 ratio ≥ 2.0. In cohort 3, pre-treatment FFPE tumor tissue was sequenced with HiSeq (Illumina) with full exome coverage for 202 genes (average depth 800) including MET. MET amplification was defined as ≥ 4 copies identified by an in-house algorithm, as previously described. [18] In cohort 4, circulating cell-free DNA (cfDNA) in plasma was analyzed by sequencing on a 54-gene platform optimized for amplifications (Guardant360^®^), using methodologies for amplification determination previously reported. [19] All testing was performed in CLIA-certified laboratories (Table 1).

The study was conducted in accordance with the guidelines of the MD Anderson Institutional Review Board.

### Statistical analysis

The primary objective was to determine prevalence of MET amplification in mCRC in tissue-based and blood-based biopsies. MET amplification rate was summarized using percentages and 95% confidence intervals (CIs). Categorical variables were compared using Fisher exact tests. All statistical tests were two-sided, and a *P* value of 0.05, when appropriate was considered significant. Statistical analyses were performed using SAS version 9.3 (SAS Institute Inc.).

## CONCLUSIONS

In this large cohort of mCRC, we observed that *de novo* MET amplification occurs rarely in mCRC, in contrast to previously reported claims. However, acquired MET amplification can be identified by cfDNA in a significant subset of mCRC patients that are refractory to anti-EGFR antibodies. We therefore, conclude that MET amplification appears to play a minor role in *de novo* colorectal carcinogenesis but may play an important role in acquired anti-EGFR resistance. These findings have clear implications for identifying patient populations and for designing appropriate clinical trials using MET inhibitors in mCRC.

## SUPPLEMENTARY MATERIALS


