# Supplementary material for: MET amplification in metastatic colorectal cancer: an acquired response to EGFR inhibition, not a de novo phenomenon
Source: Oncotarget. 2016 Jul 13;7(34):54627–31. doi: 10.18632/oncotarget.10559 (PMC5342368; doi:10.18632/oncotarget.10559)
Supplement: Supplementary file 1 [file oncotarget-07-54627-s001.pdf]

# MET amplification in metastatic colorectal cancer: An acquired response to EGFR inhibition, not a *de novo* phenomenon

## Supplementary Materials

## MATERIALS AND METHODS

### Description of cohorts

### Cohort 1 (*n* = 103)

Included newly diagnosed and previously untreated metastatic colorectal cancer (mCRC) patients with liver metastases enrolled in The Texas Medical Center Genetics (TexGen) Consortium study. In this cohort we performed analyses on the archived tissue microarrays (TMAs) prepared from resected liver metastases. MET amplification was tested using fluorescence *in-situ* hybridization (FISH).

### Cohort 2 (*n* = 208)

Included patients with mCRC, refractory to standard lines of therapy that were referred to the Department of Investigational Cancer Therapeutics. In this cohort we performed analyses on archived formalin-fixed, paraffin-embedded (FFPE) tissue from either a biopsy or surgery. These samples comprised of tumor tissue from both primary and metastatic sites. These tumor tissues were obtained prior to treatment with systemic therapies and specifically prior to any exposure to anti-EGFR antibodies (Cetuximab or Panitumumab). Met amplification was tested using FISH.

### Cohorts 3 (*n* = 279)

Included patients with mCRC, refractory to standard lines of therapy seen in the Department of Gastrointestinal Medical Oncology. In this cohort we performed analyses on archived FFPE tissue from either biopsies or surgery. These samples comprised of tumor tissue from both primary and metastatic sites. These tumor tissues were obtained prior to treatment with systemic therapies and specifically prior to any exposure to anti-EGFR antibodies (Cetuximab or Panitumumab). MET amplification was assessed using next- generation sequencing using HiSeq (Illumina) with full exome coverage for 202 genes (average depth 800), where MET amplification was defined as ≥ 4 copies identified by an in-house algorithm.

### Cohort 4 (*n* = 205)

Included mCRC patients who were treated with at least one- prior line of therapy and were enrolled on the Assessment of Targeted Therapy Against Colorectal Cancer (ATTACC) program, a molecular screening protocol. We prospectively collected plasma from these patients for circulating cell-free DNA (cfDNA) analyses. In RAS wild-type patients, these samples were collected in some patients who were refractory to anti-EGFR agents and also patients not as yet treated with these agents. Plasma samples from these patients were analyzed by Guardant sequencing platform.

**Supplementary Table S1: Mutational landscape of patients presenting with tissue MET amplifications in cohort 1–3 (Red = Mutant; Green = Wild Type; Gray = Not done)**

| Cohort* | APC | TP53 | KRAS | NRAS | PIK3CA | BRAF | EGFR |
|---|---|---|---|---|---|---|---|
| 2-Case 1 | | G245S | | | | V600E | |
| 2-Case 2 | | K132E | | | | | |
| 2-Case 3 | | 1 W91 | UNK | | | | |
| 2-Case 4 | | | G12A | | | | |
| 3-Case 1 | E1295 | R273C | | | | | |
| 3-Case 2 | | R342 | | Q61L | E542K | | |
| 3-Case 3 | | I162S | | | | | |
| 3-Case 4 | | C176W | | | | | |
| 3-Case 5 | | L130R | G12D | | | | |
| 3-Case 6 | | | | | | | |

Numbers specify the codon(s) in which mutations were present.
*Cohort 1 not included since no cases with MET amplification were seen.
Abbreviations: UNK, unknown.

**Supplementary Table S2: Acquired mutational landscape of cfDNA in patients presenting with cfDNA MET amplifications in cohort 4A after anti-EGFR therapy (Red = KRAS, NRAS, BRAF, EGFR mutations associated with anti-EGFR resistance)**

| Alterations | ≥ 1% | < 1% |
|---|---|---|
| Case 1 | FGFR2, SMAD4 | ALK, APC |
| Case 2 | SMAD4 | KIT |
| Case 3 | APC, KRAS Q61H, NRAS Q61L, SMAD4 | KRAS Q61L, ALK, APC, AR, EGFR S492R, EGFR (Others), FBXW7, KRAS G12D |
| Case 4 | TP53 | CDH1, PROC |
| Case 5 | SMAD4 | None |
| Case 6 | None | EGFR (Others) |
| Case 7 | EGFR (Others) | EGFR (Others), KRAS Q61H, KRAS G12D |
| Case 8 | None | APC |
| Case 9 | APC | PDGFRA, TP53 |
| Case 10 | APC, MYC, NF-1 | ARID1A, AR, BRCA2, FGFR1, KRAS G12A, NRAS Q61K |
| Case 11 | AR, BRCA1, NOTCH1, PIK3CA, SMAD4 | ALK, AR, BRCA1, BRCA2, FBXW7, FGFR2, FGFR3, KRAS Q61H, NF1, NOTCH1, NRAS Q61H |
| Case 12 | BRCA2, CCNE1 | AR, CDKN2A, FGFR1 |

Only discordant alterations (present in cfDNA but not in pre-treatment tissue) shown.

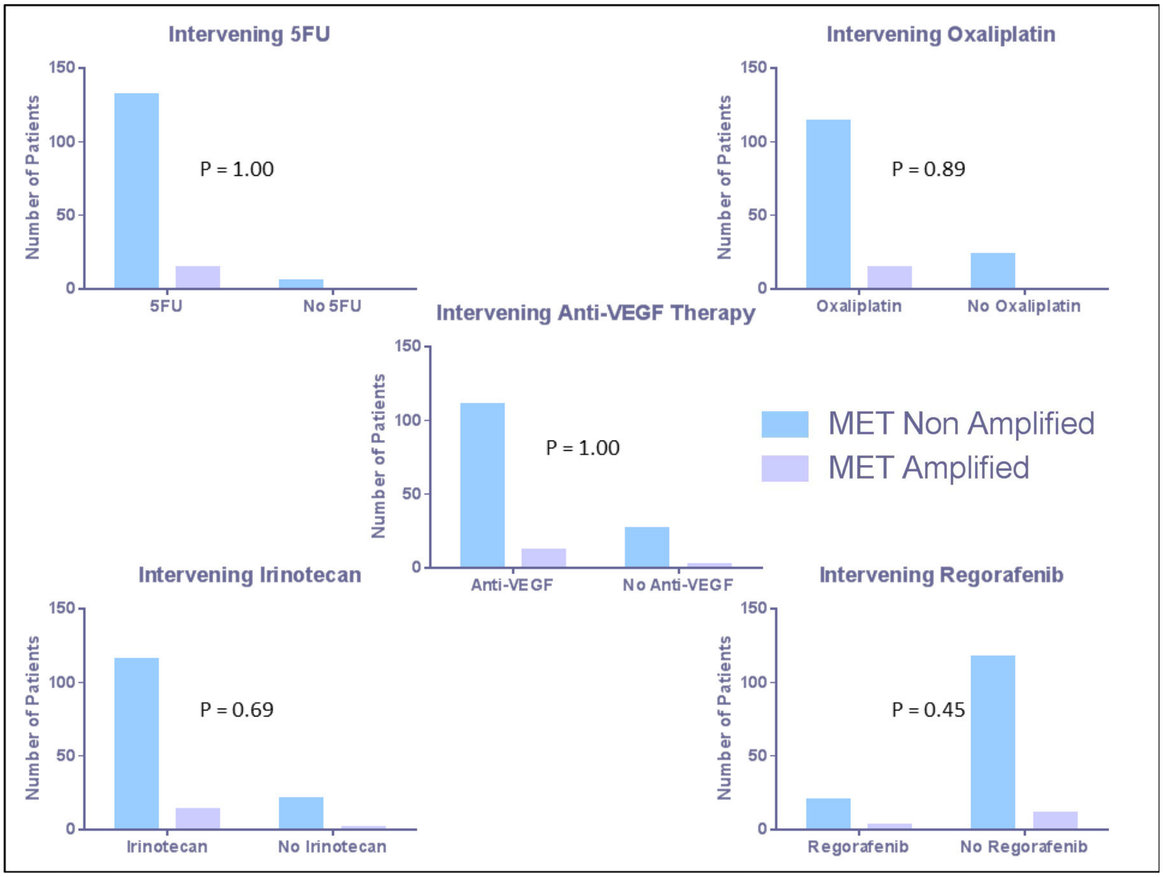


**Supplementary Figure S1: Association of cfDNA MET amplification and intervening therapies other than anti-EGFR therapy.**
